# Impact of Proliferator-Activated Receptor γ Gene Polymorphisms on Risk of Schizophrenia: A Case-Control Study and Computational Analyses

**DOI:** 10.18502/ijps.v15i4.4294

**Published:** 2020-10

**Authors:** Saman Sargazi, Fariba Mirani Sargazi, Mahdiyeh Moudi, Milad Heidari Nia, Ramin Saravani, Shekoufeh Mirinejad, Sheida Shahraki, Mansoor Shakiba

**Affiliations:** 1 Cellular and Molecular Research Center, Resistant Tuberculosis Institute, Zahedan University of Medical Sciences, Zahedan, Iran.; 2 Genetics of Noncommunicable Disease Research Center, Resistant Tuberculosis Institute, Zahedan University of Medical Sciences, Zahedan, Iran.; 3 Department of Clinical Biochemistry, School of Medicine, Zahedan University of Medical Sciences, Zahedan, Iran.; 4 Department of Psychiatry, Zahedan University of Medical Sciences, Zahedan, Iran.

**Keywords:** *Peroxisome Proliferator-Activated Receptor-γ (PPAR Gamma)*, *Polymorphism*, *rs1801282*, *rs3856806*, *Schizophrenia*

## Abstract

**Objective:** Schizophrenia (SCZ) is a common psychiatric disorder characterized by a complex mode of inheritance. Peroxisome proliferator-activated receptor-γ (PPARG) mainly regulates lipid and glucose metabolisms while it is constitutively expressed in rat primary microglial cultures. This preliminary study was aimed to investigate the relationship of two polymorphisms in the *PPARG* gene, rs1801282 C/G, and rs3856806 C/T, to the risk of SCZ in the southeast Iranian population.

**Method** **:** A total of 300 participants (150 patients with SCZ and 150 healthy controls) were enrolled. Genotyping was done using the amplification refractory mutation system polymerase chain reaction (ARMS–PCR) technique. Computational analyses were carried out to predict the potential effects of the studied polymorphisms.

**Results: **A significant link was found between genotypes of rs1801282 and SCZ susceptibility. The G allele of rs1801282 in CG and GG form of the codominant model increased the risk of SCZ by 2.49 and 2.64 folds, respectively. With regards to rs3856806, enhanced risk of SCZ was also observed under different inheritance models except for the overdominant model. Also, the T allele of rs3856806 enhanced the risk of SCZ by 3.19 fold. Computational analyses predicted that rs1801282 polymorphism might alter the secondary structure of *PPARG*-mRNA and protein function. At the same time, the other variant created the binding sites for some enhancer and silencer motifs.

**Conclusion: **Our findings showed that *PPARG* rs1821282 and rs3856806 polymorphisms associate with SCZ susceptibility. Replication studies in different ethnicities with a larger population are needed to validate our findings.

Schizophrenia (SCZ) is considered as a severe mental illness with high heritability and is characterized by hallucinations and cognitive impairment (1-3). As a complex multifactorial illness, SCZ causes disability and accounts for a significant reduction in life expectancy (4). SCZ reduces the cranial and cerebral size, which is associated with interruption of some cognitive tests, although the frontal size does not decrease (5). Disruption of the structures of the medial temporal lobe may lead to clinical symptoms that form the core of the SCZ syndrome (6). 

In this regard, disruption of the N-methyl-D-aspartate (NMDA) subtype glutamate receptor has been involved in the pathophysiology of SCZ (7). 

Peroxisome proliferator-activated receptor-γ or PPARG forms heterodimers with retinoid X receptors (RXRs) and functions as ligand-dependent transcription factors (8). Besides, the protein encoded by this gene, PPARG, modulates adipocyte differentiation (9). The gene encoded by this protein, *PPARG*, is up-regulated in lipid-aggregation macrophages of the coronary artery (10).

PPARG has been involved in inhibiting the expression of anti-inflammatory cytokines and induced enzymes, such as cyclo-COX-2 (COX-2) (11). In macrophages, PPARG ligands suppress the expression of toll-like receptor (TLs)-mediated genes, via a "ligand-dependent transrepression" mechanism (12). During inflammatory processes, PPARG ligands down-regulate cell adhesion molecules on endothelial cells and decrease chemokines secretion, and therefore, impairs leukocytes recruitment into inflammatory sites (13). On the other hand, PPARG controls glucose/lipid metabolism (10, 14). There is a hypothesis suggesting that the emergence of SCZ-like syndrome contributes to actions exerted by retinoid X receptor-α/PPARG heterodimers (15). Based on this hypothesis, single-nucleotide polymorphisms (SNPs) located in the *PPARG* gene might affect the performance of the retinoid cascade, and therefore, have a profound effect on SCZ etiology (15). 

Several SNPs in the *PPARG* gene have been discovered, and rs1801282 (Pro12Ala), with a minor allele frequency (MAF) of 0.07 (based on information from 1000 genome project), is the most studied one (16, 17). Another polymorphism located in *PPARG*, rs3856806 (His449His), is a frequent *PPARG* synonymous/3′-untranslated region (3′-UTR) polymorphism (MAF = 0.127) (17).

Studies have indicated that SCZ is affected by SNPs in candidate genes (18). In this preliminary study, we aimed to investigate the impact of two polymorphisms in the *PPARG* gene, rs1801282, and rs3856806 C/T, on the risk of SCZ among a population in southeast of Iran.

## Materials and Methods

***Population sample***

A total of 300 people (150 SCZ patients and 150 healthy controls) were enrolled. Diagnosis of SCZ was made according to DSM–V operational diagnostic criteria (19). Patients with mood disorders, mental retardation, and history of drug or alcohol abuse were excluded. Healthy subjects had no history of substance abuse or any neuropsychiatric disorder. The local ethical review board approved the study (IR.ZAUMS.REC.1398.136). Written information regarding the try-out was given to all participants. For isolation of DNA, 5 mL of whole venous blood was withdrawn and collected in conventional EDTA tubes.

***Genotyping***

**Figure 1:**
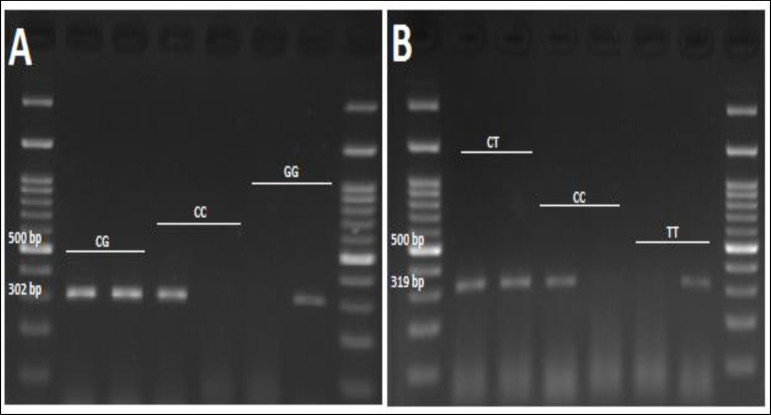
Genotyping of A: rs1801282 and B: rs3856806 Variants in *PPARG* Gene via ARMS-PCR Method

Table 1 shows the information of the studied polymorphisms. The *PPARG* gene is located in the chromosomal region of 3p25.19 (20). Two polymorphisms in the *PPARG* gene, with minor allele frequency higher than 0.05, were selected. Genotyping was carried out via the amplification refractory mutation system polymerase chain reaction (ARMS-PCR) technique, using primers shown in Table 2. In each tube, 0.15 mL of PCR reaction, 0.6 μL (90 ng) of isolated DNA, 0.5 μL of each primer (Cinnaclon, Tehran, Iran), 6.4 mL of dH2O, and 7 mL of Taq 2x master mix (Parstous, Iran) were added. The cycling conditions consisted an initial denaturing at 94 °C for 6 min; 35 cycles at 94°C for 30 s; at 60°C (for rs1801282) and 59°C (for rs3856806) for 30 s, and 72 °C for 40 s, and a final extension at 72°C for 6 min. The amplified products were electrophoresed on 2% agarose gel containing DNA safe stain (SinaClon, Iran), and DNA bands were visualized under UV light (Figure 1). For C or G allele of rs1801282, the fragment size was 302 bp. In the meantime, a 319 bp amplicon represented either T or C allele of rs3856806.

***Computational Analyses***

Several Bioinformatics tools were employed to examine the possible effects of rs1801282 and rs3856806 polymorphisms. SNAP database was used to predict the effect of non-synonymous polymorphisms on the function of PPARG protein using submitted protein information (21). Chou–Fasman database was used to examine the impact of the SNPs in *PPARG*-mRNAs and the secondary structures of this protein. Chou–Fasman algorithm predicts the effect of each amino acid on the locations of alpha-helix and beta-strand in a given sequence (22). Splice Aid2 database was used to determine splicing pattern alteration for rs3856806 polymorphism (23). Finally, the WebLogo database was used to illustrate the conservation of the polymorphisms between different organisms (24).

***Statistical Analysis***

For data analysis, SPSS software version 22 (Chicago, Illinois, USA) was used. Alleles and genotypes of the studied SNPs were compared using the chi-square test. The odds ratios (ORs) and 95% confidence intervals (95% CI) were also calculated. The SNPAnalyzer2 software was used to calculate genotype and allele frequencies. P-values < 0.05 were considered significant.

## Results

The mean age of the studied population was 36.51 ± 10.52 in cases and 36.38 ± 10.89 in controls. We did not find any significant differences among the studied groups in age (p = 0.80) and sex (p = 0.91). 

Our findings revealed that the G allele of rs1801282C/G in CG and GG forms of the codominant model increased SCZ risk by 2.49 and 2.64 folds (OR = 2.49, 95% CI = 1.38 - 4.54, p = 0.00, and OR = 2.64, 95% CI = 1.34 - 5.21, p = 0.00, respectively). For rs1801282C/G, enhanced risk of SCZ was under dominant (CC vs. GG+GC), recessive (GC+CC vs. GG), and overdominant (GG+CC vs. CG) contrasted models (Table 3). With regards to rs3856806, the T-allele conferred an increased risk of SCZ by 3.19 folds. Besides, the T-allele under codominant TT vs. CC, dominant (CC vs. TT+TC), and recessive (CC+CT vs. TT) genetic models enhanced SCZ susceptibility by 5.11, 2.96, and 4.96 folds, respectively (Table 4). 

The interaction analysis of rs1801282 and rs3856806 genotypes indicated that the frequency of CC/CC combination was higher in controls; therefore, it was selected as the reference. As shown in Table 5, the CC/TT, GG/CC, and GG/TT genotypic combinations markedly enhanced the risk of SCZ (OR = 5.07, OR = 2.36, OR = 7.33, respectively). The result of haplotype analysis is shown in Table 6. In this subject, all 3 haplotypes (CT, GC, and GT) significantly enhanced SZN susceptibility. In our study, the amount of D' coefficient was 0.06, which means these two polymorphisms are not in linkage disequilibrium (data not shown).

**Figure 2:**
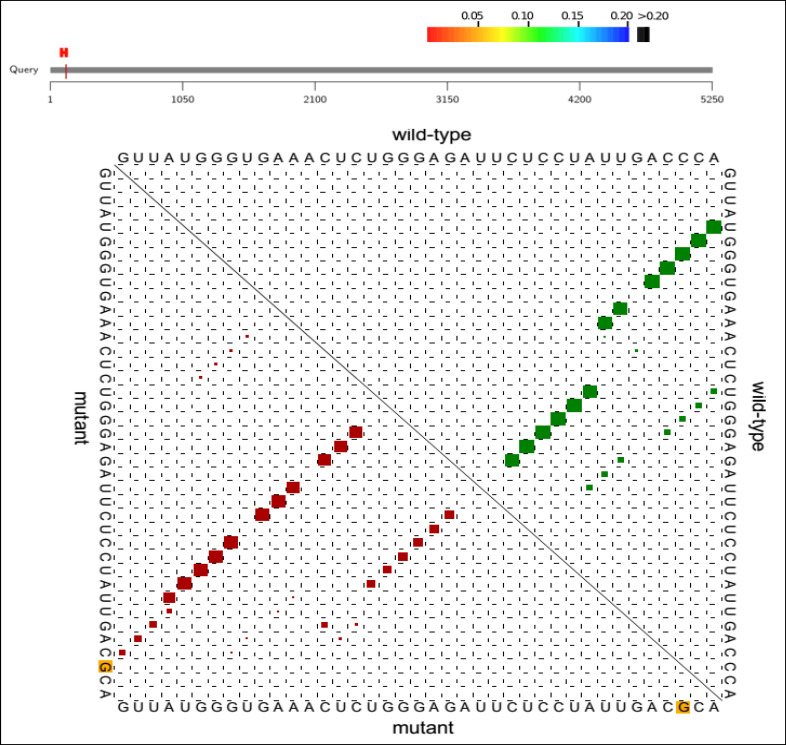
The Potential Effects of rs1801282 Polymorphism on the Secondary Structure of *PPARG*-mRNA

**Figure 3:**
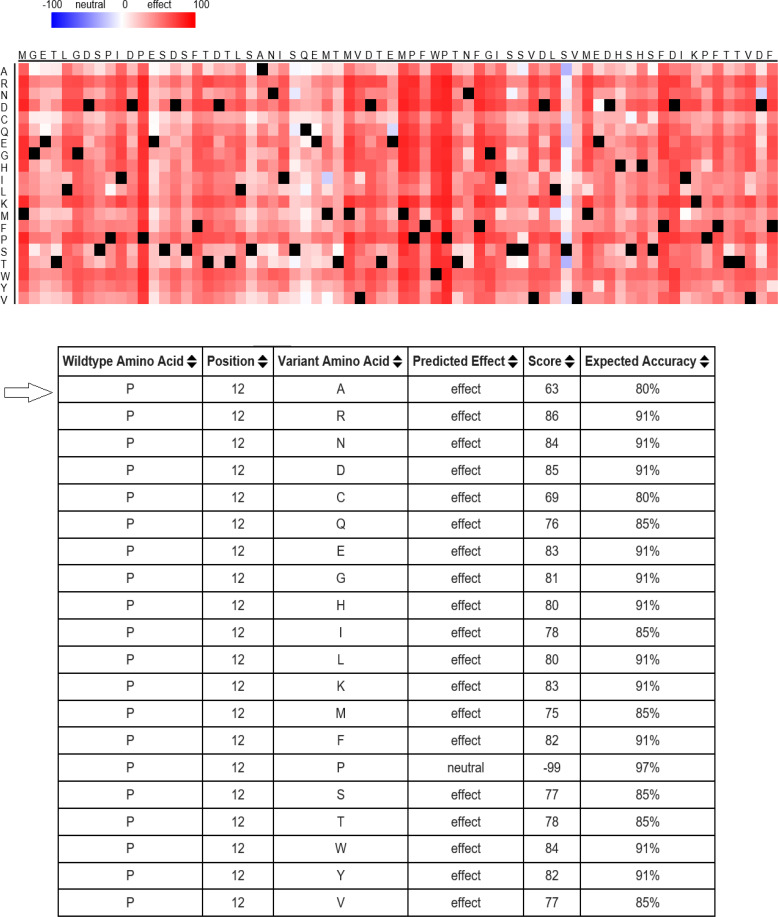
Potential Effect of Pro12Ala Substitutions on PPARG Protein Function Evaluated by SNAP

**Figure 4:**
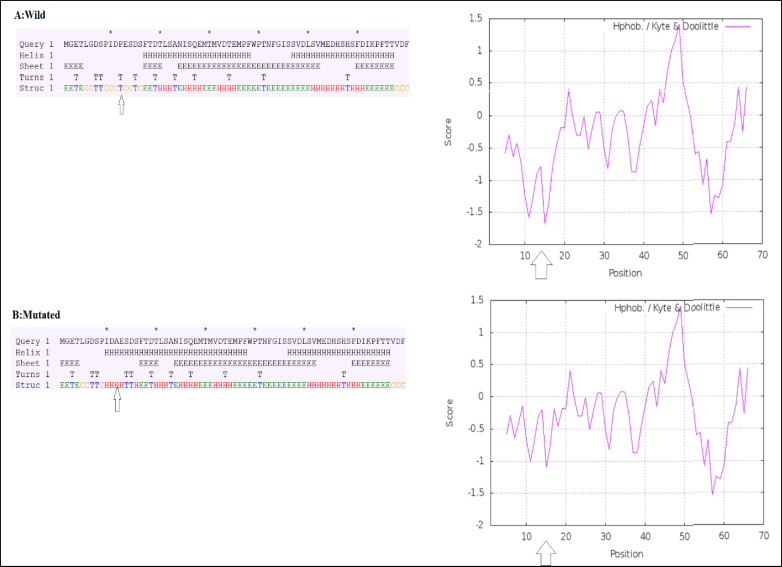
Prediction of Hydrophobicity and Secondary Structures. A & A′: Hydrophobicity Plot for P12A Phenotype; B & B′: Chou–Fasman's Secondary Structure for Pro12Ala. The Residue 12 Is Shown by an Arrowhead

**Figure 5:**
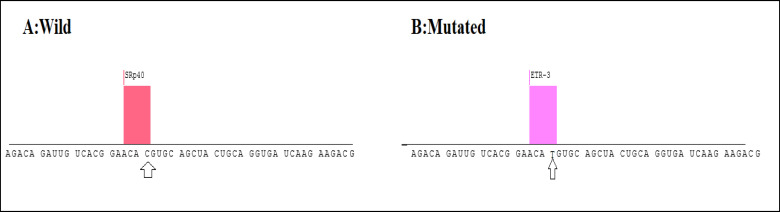
Using the Splice Aid2 Database to Predict the Possible Effects of rs3856806 Polymorphism on Gene Splicing. The SRp40 Motif Was Created by the C Allele of This Variant, While the ETR-3 Motif Was Created by the T Allele

**Figure 6:**
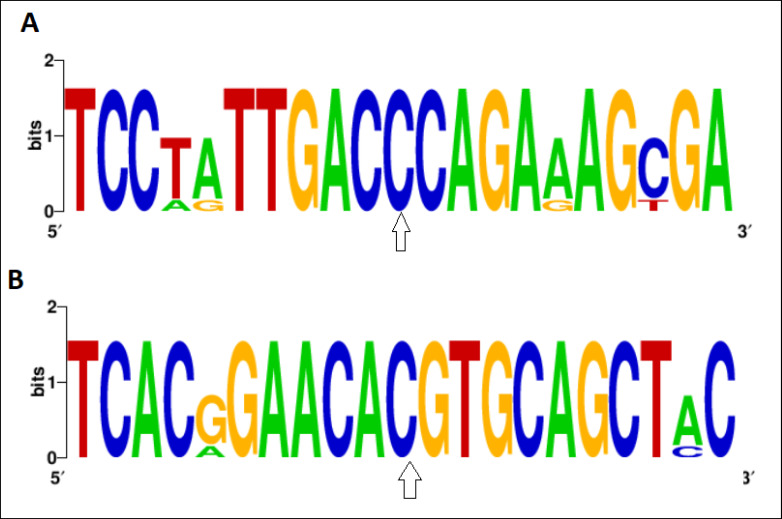
Weblogo Database Was Used to Compare the Conservation of A: rs1801282 and B: rs3856806 Polymorphisms between the Different Organisms. The Two Variants of the *PPARG* Gene Has High Genetic Conservation between Human and Other Primates

Computational analyses demonstrated that the C→G conversion of rs1801282 polymorphism leads to a proline-alanine substitution at codon 12. Likewise, the rs3856806C/T polymorphism leads to a C→T conversion at codon 449 of the *PPARG* gene (a histidine-histidine substitution). Predicting *PPARG* rs1801282 eﬀects the secondary structure of *PPARG*-mRNA and showed that this polymorphism results in fundamental changes in the secondary structure of mRNA (p-value = 0.002) (Figure 2). As shown in Figure 3, the SNAP database predicted a signiﬁcant eﬀect of the Pro12Ala substitution on the protein structure of PPARG (Score: 63; Expected accuracy: 80%). Moreover, the Chou–Fasman hydrophobic score of rs1801282 at position 12 was -0.75 for proline compared to -0.3 for alanine residue (Figure 4). The Splice Aid2 database was used to predict the possible effects of rs3856806 polymorphism on gene splicing. We found that the SRp40 motif was created by the C allele of this variant, while the ETR-3 protein motif was created by the T allele (Figure 5). Conservation of s3856806 and rs1801282 SNPs was also illustrated by the WebLogo tool, indicating that these variants are located in a well-conserved region across humans and other primates (Figure 6).

**Table 1:** Information of the Studied Polymorphisms

***PPARG*** ** polymorphisms**	**Chromosome**	**Functional Consequence**	**Chromosome ** **position**	**Allele ** **(major/minor)**	**Aminoacid ** **Exchange**
rs1801282	3	Missense variant	3	C/G	P/A
rs3856806	3	Synonymous variant, 3’ UTR variant	3	C/T	H/H

**Table 2:** Primers Used for Genotyping of SNPs within the *PPARG* Gene

**SNP**	**Primers**	**Sequence (5′ to 3′)**	**Annealing ** **Temperature**	**Length of PCR ** **product** **(bp)**	
	Forward (C-allele)	CTGGGAGATTCTCCTATTGAAC			302
rs1801282	Forward (G-allele)	CTGGGAGATTCTCCTATTGAAG	60 °C		302
	Reverse (common)	GGAAATGGGATCCATGCACAG			302
	Forward (C-allele)	TCAGACAGATTGTCACGGAACTC			319
rs3856806	Forward (T-allele)	TCAGACAGATTGTCACGGAACTT	59°C		319
	Reverse (common)	CTATCAGCAATTTCATAATATGGT			319

**Table 3:** Allelic and Genotypic Distribution of *PPARG* rs1801282 Polymorphism in SCZ Cases and Healthy Controls

**Model**	**SCZ (n)**	**Control (n)**	**OR (95%CI)**	**P-value**
Codominant				
CC	85	109	1	
CG	41	21	2.49 (1.38-4.54)	0.00
GG	31	15	2.64 (1.34-5.21)	0.00
Allele				
C	211	239	1	
G	103	51	1.84 (0.97-3.49)	0.06
Dominant				
CC	85	109	1	
GG+GC	72	36	2.56 (1.56-4.19)	0.00
Recessive				
GC+CC	126	130	1	
GG	31	15	2.12 (1.10-4.14)	0.01
Over-dominant				
GG+CC	116	124	1	
GC	41	21	2.09 (1.15-3.73)	0.00

SCZ: schizophrenia, OR: odds ratio, CI: confidence interval, Boldface shows statistical significance (P<0.05).

**Table 4:** Allelic and Genotypic Distribution of *PPARG* rs3856806 Polymorphism in SCZ Cases and Healthy Controls

**Model**	**SCZ (n)**	**Control (n)**	**OR (95%CI)**	**P-value**
Codominant				
CC	87	114	1	
CT	27	20	1.77 (0.92-3.35)	0.08
TT	43	11	5.11 (2.50-10.51)	0.00
Allele				
C	201	248	1	
T	113	42	3.19 (1.61-6.32)	0.00
Dominant				
CC	87	114	1	
TT+TC	70	31	2.96 (1.77-4.90)	0.00
Recessive				
CC+CT	114	134	1	
TT	43	11	4.96 (2.25-9.32)	0.00
Overdominant				
CC+TT	130	125	1	
CT	27	20	1.30 (0.68-2.42)	0.41

SCZ: schizophrenia, OR: odds ratio, CI: confidence interval, Boldface shows statistical significance (P<0.05).

**Table 5:** Interaction of *PPARG* rs3856806C/T and rs1801282C/G Polymorphisms on SZN Risk

**rs1801282C/G**	**rs3856806C/T**	**SCZ (%)**	**Control (%)**	**OR (95%CI)**	**P-value**
CC	CC	49 (31.2%)	83 (57.2%)	1	
CC	CT	12 (7.6%)	18 (12.4%)	1.13 (0.49-2.53)	0.77
CC	TT	24 (15.3%)	8 (5.5%)	5.07 (2.12-12.18)	0.00
GC	CC	24 (15.3%)	21 (14.5%)	1.94 (0.98-3.84)	0.06
GC	CT	10 (6.4%)	0 (0.0)	-	
GC	TT	6 (3.8%)	0 (0.0)	-	
GG	CC	14 (8.9%)	10 (6.9%)	2.36 (0.98-5.75)	0.04
GG	CT	5 (3.2%)	2 (1.4%)	4.23 (0.78-22.65)	0.06
GG	TT	13 (8.3%)	3 (2.1%)	7.33 (1.91-27.03)	0.00

SCZ: schizophrenia, OR: odds ratio, CI: confidence interval, Boldface shows statistical significance (P<0.05).

**Table 6:** Haplotype Analysis of *PPARG* Gene Polymorphisms between SCZ Cases and Healthy Controls

**rs1801282C/G**	**rs3856806C/T**	**SCZ**	**Control**	**OR (95%CI)**	**P-value**
C	C	0.41	0.73	1	
C	T	0.26	0.13	3.56 (1.65-7.67)	0.00
G	C	0.23	0.09	4.55 (1.92-10.76)	0.00
G	T	0.10	0.05	3.56 (1.14-11.13)	0.02

SCZ: schizophrenia, OR: odds ratio, CI: confidence interval, Boldface shows statistical significance (p<0.05).

## Discussion

The current study assessed the link between two polymorphisms of the *PPARG* gene with susceptibility to SCZ. Our results indicated that the rs3856806 polymorphism correlated with the risk of SCZ under codominant, dominant, and recessive models. Also, we noticed that the T-allele of rs3856806 significantly enhanced SCZ vulnerability in our population. Likewise, the other *PPARG* gene polymorphism, rs1801282, was associated with SCZ susceptibility under all the assessed genetic models, except for the allelic contrast model.

PPARG receptor, encoded by the *PPARG* gene on chromosome 3, acts as a receptor and affects some downstream molecules (25). PPARG receptors are essential molecules involved in fat storage (26), adipose tissue metabolism, insulin sensitivity, and inflammation responses (27-29). Besides, the altered glucose levels are reported in SCZ patients treated with antipsychotics (15). Therefore, the alterations of the PPARG receptor affect glucose level, which subsequently influences the psychosis profile in SCZ patients exposed to antipsychotics. Few studies have also reported the relationship between *PPARG* polymorphisms and the risk of obesity or psychiatric disorders in patients treated with olanzapine or clozapine (30, 31). To this date, no report is available regarding the association between *PPARG* polymorphisms with SCZ risk. The most relevant study was conducted by Liu et al. in 2014, who found that rs3856806 in exon 6 of the *PPARG* gene was associated with psychosis profile and glucose levels in SCZ patients receiving treatments (15). Herken et al. showed that SNPs in the *PPARG* gene might contribute to olanzapine-induced weight gain and the selection of antipsychotic medication for SCZ patients (30). In 2009, Ben Ali et al. reported that the Pro12Ala polymorphism is correlated with obesity risk in nondiabetic men from Tunisian origin (31). In contrast, it has been reported that rs1801282 polymorphism plays no critical role in antipsychotic drug-related weight gain (32). However, information about these gene polymorphisms is still contradictory, which may be due to differences in genetic background between studied populations.

In our study, we examined the possible link between 2 polymorphisms of the *PPARG* gene and SCZ risk. Haplotype and interaction analyses showed that some genotype combinations and haplotypes might increase the risk of SCZ. Finally, computational studies proposed that the rs1801282 polymorphism may alter the secondary structure of *PPARG*-mRNA and protein function. At the same time, the other variant created the binding sites for some enhancer and silencer motifs. These findings speculated that the genetic variations in the *PPARG* gene might affect its function. 

## Limitation

There were a few limitations in the present study, including long-term antipsychotic treatments, different environmental factors related to the etiology of SCZ, and various ethnic groups living in the southeast of Iran. Moreover, our sample size was relatively small.

## Conclusion

Our findings indicated that *PPARG* rs1821282 and rs3856806 polymorphisms are involved in the etiology of SCZ. Using bioinformatics tools, we predicted that the variants might have possible effects on the function of PPARG protein in SCZ patients. Discovering the biological impact of the PPARG in the brain may help to find molecular mechanisms underlying such genetic associations.
